# Use of Sirolimus, an mTOR Inhibitor, to Treat Sarcoidosis in Multiple Systems

**DOI:** 10.1007/s12265-025-10700-4

**Published:** 2025-09-25

**Authors:** Liam McGuire, Roxxy Brown, Angeliki Asimaki

**Affiliations:** 1 Cardiovascular and Genomics Research Institute, School of Health & Medical Sciences, City St. George’s, University of London, Cranmer Terrace, SW17 0RE London, UK; 2 Strata Medical Research, North Miami, Florida, USA

**Keywords:** Sarcoidosis, Sirolimus, mTOR

## Abstract

Sarcoidosis is a heterogenous, multi-systemic granulomatous disease with highly variable incidence (5–40/100,000) and a disproportionate mortality burden in those presented with pulmonary fibrosis or cardiac involvement. Yet, current management strategies are symptom-targeting, not always effective and come with significant side effects. Preclinical murine models of sarcoidosis have shown that aberrant mTORC1 activation promotes macrophage-driven inflammation and disrupts autophagic clearance, sustaining granuloma formation. Sirolimus, a selective mTORC1 inhibitor, restores autophagy and macrophage function, offering a targeted therapeutic approach. Herein, we present the first comprehensive review of all known clinical cases of sirolimus use in different forms of sarcoidosis. All studies reviewed suggest that sirolimus may be an effective, yet safe, mechanism-targeting therapy for patients with sarcoidosis not responding to conventional pharmacological interventions.

**Graphical Abstract:**

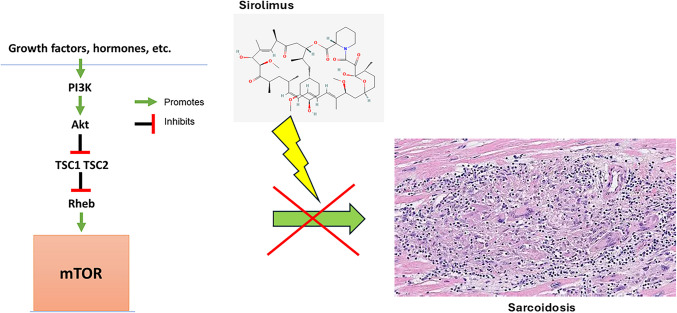

## Introduction

Sarcoidosis is a multi-systemic, immune-mediated inflammatory disease characterized by the accumulation of granulomas in multiple organs throughout the body. Granulomas are defined as compact collections of lymphocytes and macrophages that have differentiated into epithelioid cells, and can cause fibrosis, scarring and organ failure [1, 2]. The precise aetiology of sarcoidosis is largely unknown; certain case reports have identified mutations in genes involving mammalian target of rapamycin (mTOR) signalling and autophagy, however the underlying pathology of most of the cases remains unresolved [1–4].

Sarcoidosis is highly heterogenous. Although it affects the lungs in up to 90% of cases, it can also affect the lymphatic system, skin, heart and larynx, and can develop in the kidney and liver post organ-transplantation [5]. Cardiac sarcoidosis (CS) is characterized by inflammatory granulomatous infiltration in the heart, progressive interstitial, perivascular fibrosis, and diastolic dysfunction with preserved ejection fraction (EF). It is life-threatening if diagnosed late and left untreated, and can lead to cardiac fibrosis, arrhythmias and sudden cardiac death (SCD) [3].

Although cardiac involvement has only been reported in 5% -7% of systemic sarcoidosis cases, at postmortem evaluation, the heart appears to be affected in 25%-58% of cases [3]. Patients with systemic sarcoidosis and CS have a worse prognosis than patients without cardiac involvement. Patients with heart failure and reduced EF in particular carry a poor prognosis, with a 10-year survival of 19%-53% without cardiac transplantation [6].

The prevalence of sarcoidosis varies greatly across the world, from 1–5 per 100,000 in parts of Asia to 140–160 per 100,000 in Sweden and Canada [7]. In the United States, non-Hispanic Black patients show the highest incidence and prevalence of sarcoidosis [7], particularly Black American women, where the incidence from 1995 – 2007 was 71 per 100,000 [8]. Social and environmental factors play an important role in the development of sarcoidosis. Systemic and structural racism in the U.S. has been linked to increased rates of sarcoidosis in Black populations, where poor housing and working conditions can increase exposure to potential sarcoidosis triggers [7]. Furthermore, following the World Trade Centre disaster in 2001, there was a significant increase in sarcoidosis-like granulomatous pulmonary disease among New York Fire Department rescue workers compared to non-exposed individuals [1].

### Current Therapeutic Landscape

There is currently no cure for sarcoidosis, and the current range of available treatments only aim to control symptoms [3]. Typically, first-line treatment is corticosteroids, primarily prednisone and its derivative, prednisolone [9]. Corticosteroids are used to treat a broad range of autoimmune, allergic and inflammatory disorders such as asthma, chronic obstructive pulmonary disorder, rheumatoid arthritis and Crohn’s Disease [10]. They are synthetic analogues of naturally occurring steroid hormones produced by the adrenal cortex, and include the glucocorticoids, which have immunosuppressive and anti-inflammatory effects [10].

First-line agents are often combined with steroid-sparing second-line agents such as methotrexate, azathioprine, leflunomide and mycophenolate [1, 9–12]. These agents, although acting as immunosuppressives, have less adverse effects compared to corticosteroids [9]. Methotrexate is the most prescribed second-line agent [9, 11], and induces autophagy via nucleic acid and amino acid synthesis inhibition [1, 9]. 20–40% of patients don’t respond to methotrexate, and treatment can take up to 6 months before a response is seen [9, 11]. Azathioprine, upregulates autophagy by reducing mTORC1 signalling [1]. It has similar efficacy to methotrexate and has been shown in multiple studies to be an effective steroid-sparing agent [9, 11].

Third-line agents are also available, such as infliximab and adalimumab, tumour necrosis factor alpha (TNF-a) inhibitors, which have all been shown to be effective in clinical trials [13, 14]. In one study, where 26 multi-organ sarcoidosis patients received infliximab over a prolonged course of therapy (up to 85 months), 58.5% showed sustained resolution or improvement [15]. However, their effectiveness remains somewhat unconvincing. A recent Cochrane Review found that patients treated with adalimumab and infliximab had significantly higher numbers of total adverse events compared to controls [16]. Another study reported that 90 patients receiving different anti-TNF-a therapies (etanercept: 59%, adalimumab: 23% and infliximab:18%) all developed sarcoidosis-like lesions which was attributed to anti-TNF-a therapy [17]. Calcineurin inhibitors like cyclosporine and tacrolimus are also used post-organ transplantation [2].

### Limitations of Current Management Options

Sarcoidosis is a chronic disease that requires prolonged therapy; therefore, it is critically important to consider the long-term adverse effects of potential treatment plans. Multiple lines of evidence show that prolonged treatment with corticosteroids, as well as exposure to high doses, have a broad range of negative side effects [3, 9–11]. Adverse side effects can be seen in up to 90% of patients who take corticosteroids for more than 60 days. These range from minor cases of acne to Cushing syndrome in more severe cases, which if untreated, can lead to diabetes mellitus and potentially life-threatening heart disease. Chronic use of glucocorticoids can lead to osteoporosis and bone fractures, psychiatric issues (mood disorders, anxiety, delirium, panic disorder), myopathic issues (muscle weakness, atrophy) and cardiovascular effects such as hypertension, hyperglycaemia and obesity [10, 18]. These adverse effects are also seen at lower doses (suggested dose range of 20-40 mg/day initially before tapering to 7.5-15 mg/day, with lower doses being ≤ 7.5 mg/day) [9, 18].

Careful monitoring of patients about to start, or already on corticosteroid regimes, is necessary. A history and physical exam are required to assess risk factors and preexisting conditions which could be worsened by corticosteroids [10]. Considering the prevalence of bone fractures in patients treated with corticosteroids, it is necessary for clinicians to consider bone mineral density (BMD) testing at baseline, and again 1-year post-treatment initiation [10], as well as height measurement and screening for fragility fractures.

Iatrogenic illness is another factor to consider with corticosteroid therapies. Should a complication occur, patients must gradually taper from such a treatment, as rapid and complete withdrawal can cause adrenocorticotropic hormone suppression and flare-ups of the underlying disease [10]. Furthermore, long-term high-dose treatment with corticosteroids can lead to suppression of the hypothalamic–pituitary–adrenal (HPA) axis, which promotes development of Addison disease [10, 18]. This HPA suppression can last for as long as 9–12 months following withdrawal [10]. Clinicians must carefully consider the implications of the effect of corticosteroids on the HPA axis, and the length of taper is dependent on how long a patient has been on the medication [18].

Toxicity in the liver, kidney or bone marrow has also been associated with steroid-sparing agents and is highly challenging to measure [9, 11]. Gastrointestinal side effects have also been reported, with weight gain attributed to corticosteroid use, nausea and diarrhoea to methotrexate and vomiting and weight loss to azathioprine and mycophenolate [9].

Third-line treatments such as TNF-a inhibitors also can cause significant adverse effects, the most common being severe infections. Infections include bacterial, fungal, viral or atypical infections, and these may be fatal. They are more common in patients receiving first- or second-line agents in combination with anti-TNF agents [19]. *Russell *et al. reported that 57.7% of patients treated with infliximab suffered adverse effects including minor infection, rash and pneumonia as well as sepsis [15]. Three patients (12%) had to permanently discontinue treatment after they developed recurrent sinusitis, positive tuberculosis skin test and sever pneumonia respectively [15].

In light of the aforementioned adverse effects, the need to develop mechanism-based therapies for sarcoidosis is pressing. This review synthesizes evidence on the effectiveness and safety of the mTOR inhibitor sirolimus in varying forms of sarcoidosis.

### Methods

**Fig. 1:**
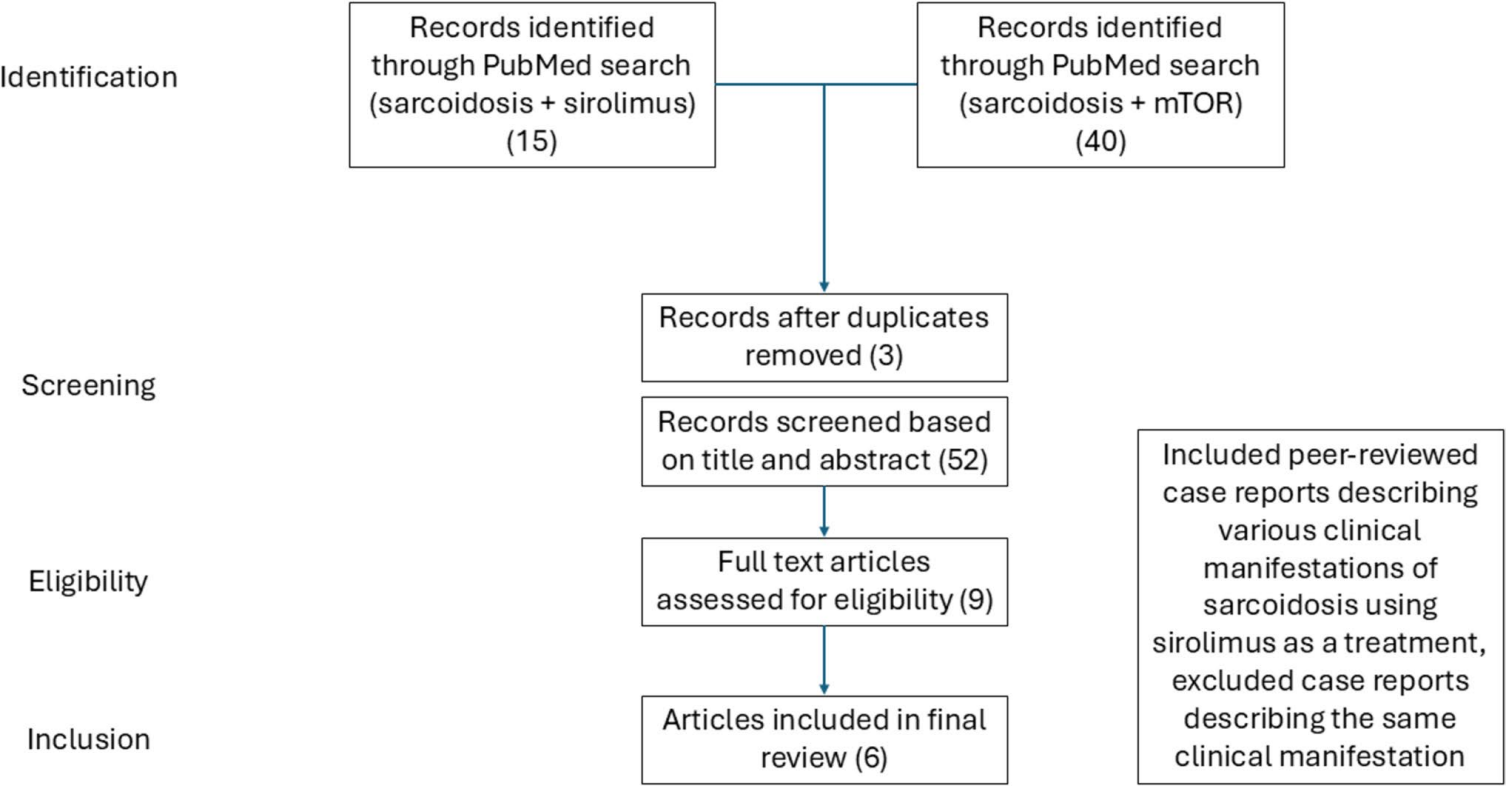
PRISMA-style flow diagram of study selection protocol: This diagram illustrates the study selection process, showing the number of records identified, screened, assessed for eligibility, and included in the final review

A PRISMA-style flow diagram describing literature search methodology can be found in Fig. 1. Pubmed was used as a database to search for literature selection. Peer-reviewed case reports describing different clinical manifestations of sarcoidosis and sirolimus were included. The key works used to search were [sarcoidosis + sirolimus] and] sarcoidosis + mTOR].

### Rationale for mTOR Inhibition in Sarcoidosis

**Fig. 2:**
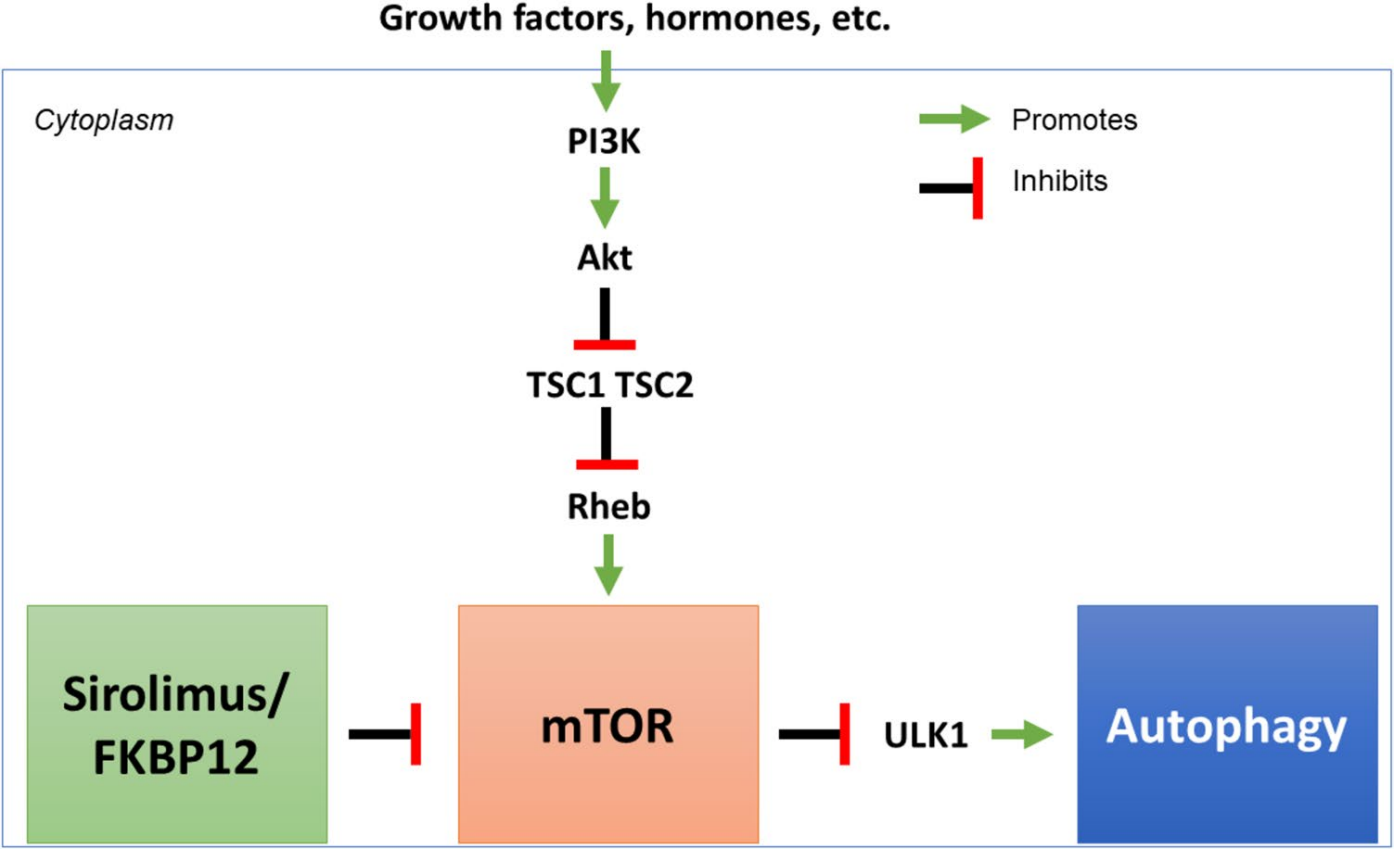
mTOR/autophagy pathway: Sirolimus inhibits mTOR, leading to increased autophagy. PI3K phosphinositide 3-kinase, Akt serine/threonine kinase, TSC1/2 tuberous sclerosis complex 1/2, Rheb Ras homolog enriched in brain, ULK1 Unc-51-like autophagy-activating kinase [1, 24, 27]

mTORC1 is an autophagy suppressor that regulates the initiation step of autophagy [1]. The mTORC1 pathway has been shown to be involved in granuloma formation and is targetable for developing mechanism-based therapies [6]. Whole-exome sequencing (WES) analysis of familial forms of sarcoidosis has suggested that genetic variants encoding regulators of mTOR and autophagy-related proteins can be disease-causing [1]. This defect in autophagy decreases the clearance of infectious agents and results in granuloma formation [1]. mTOR is negatively regulated upstream by tuberous sclerosis complex 1/2 (TSC1-TSC2) [1]. The mTOR pathway is depicted in Fig. 2 [1, 20, 21].

It was previously shown that deletion of TSC2 in myeloid lineage Lyz2 expressing cells led to the spontaneous development of pulmonary sarcoid-like granulomas in mice. This conditional myeloid deletion of Tsc2 resulted in the formation of non-caseating granulomatous aggregates in the lung and liver, but also in the lymph nodes of 3-month-old mice. By 4 months these mice developed swollen paws and tails caused by excessive granulomas and they also showed hypertrophic peritoneal macrophages. The mice also had a population of hypertrophic lung cells comprising hypertrophic alveolar macrophages but no other known myeloid or lymphoid immune cells [22]. Recently, the Weichhart group generated a mouse with Cre-recombinase-mediated conditional deletion of the tuberous sclerosis 2 (TSC2) gene in CD11c + cells (TSC2fl/flCD11c-Cre; termed TSC2ko) [3]. These mice showed spontaneous development of sarcoid-like granulomas in the lungs [3]. Subsequently, the cardiac phenotype of mice with chronic activation of mTORC1 signalling in myeloid cells was investigated. The hearts of TSC2ᴷᴼ mice exhibited increasing numbers of inflammatory infiltrates, particularly in the base and midventricular region of the left ventricle, septum, papillary muscles, and to a lesser extent, atria and pericardium. These infiltrates were predominantly Mac-2⁺ macrophages expressing CD68 and CD206, found interstitially and perivascularly [3]. In older animals (~ 54 weeks), structures resembling non-necrotizing granulomas and giant cells appeared, indicating progressive severity. The hearts also exhibited significant interstitial and perivascular fibrosis, which worsened with age. Finally, signal for the desmosomal protein plakoglobin and the major ventricular gap junction protein connexin 43 (Cx43) was significantly depressed at cardiac intercalated disks, as seen in the human disease [23]. Cx43 remodelling is a known substrate for life-threatening arrhythmias [23].

Treatment of this mouse model with the mTOR inhibitor everolimus resolved granulomatous infiltrates, prevented fibrosis development and significantly improved cardiac function. Furthermore, mTOR signalling was detected in CD68 + macrophages in 78% of hearts from SCD victims diagnosed with CS postmortem, indicating involvement of the mTOR pathway in the pathogenesis of several forms of sarcoidosis [3]. BAY11-7082, a nuclear factor kappa beta (NF-κB), rescues the disease phenotype in in vitro and in vivo models of arrhythmogenic cardiomyopathy, a known phenocopy of sarcoidosis [23, 24]. Yet, the NF-κB inhibitor did not improve cardiac symptoms in the mTORC1-dependent mouse model for CS [3].

**Fig. 3:**
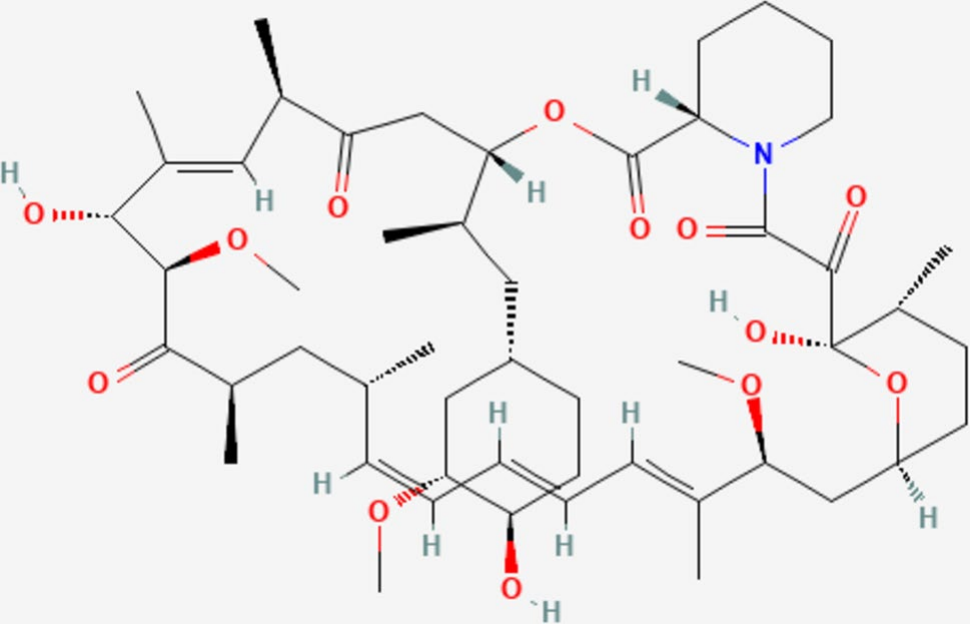
2D Chemical structure of sirolimus. PubChem Identifier: CID:5,284,616. URL: https://pubchem.ncbi.nlm.nih.gov/compound/5284616#section=2D-Structure

The prototypical and most widely recognized mTOR inhibitor is sirolimus, a macrocyclic triene antibiotic (PubChem CID:5,284,616). Its chemical structure is depicted in Fig. 2. It was first extracted from *Streptomyces hygroscopius* in 1972 [25, 26]. It was later assigned the United States Adopted Names Council designation Sirolimus and the trade mark name Rapamune [27]. Sirolimus is an important drug in oncology, cardiology and transplantation medicine [28]. Shortly after its initial extraction, its potential as an effective immunosuppressor and anti-cancer drug was unravelled, as well as its effectiveness post-organ transplantation [20]. Sirolimus acts on lymphocytes through the mTOR pathway, arresting the cell cycle in the G1-S phase, preventing cell cycle progression and proliferation, which can lead to the development of granulomas [1, 29]. Specifically, it interacts with the FK506-binding protein 12 (FKBP12), inhibiting the formation of mTORC1 [25]. Accordingly, its mechanism of action differs from that of its structural analogue tacrolimus, a calcineurin inhibitor, which inhibits the progression of T cells from G0 to G1 [29] (Fig. 3).

Sirolimus was first FDA-approved in 1999 for immunosuppression post-organ transplantation. Over the next decade, many breakthroughs further demonstrated the potential applications of sirolimus as a mechanism-based therapeutic through interaction with the mTOR pathway [20]. Throughout the 2010’s, various clinical trials involving sirolimus and its derivatives have shown promise, with the SUCCEED clinical trial for sarcoma leading to the FDA approval of ridaforolimus ( a synthetic analog of sirolimus) in 2011, as well as further demonstrations of sirolimus’s anti-cancer effectiveness. In more recent years, nab-sirolimus has been FDA approved for PEComa, a soft tissue cancer affecting women, as well as the development of mTOR inhibitors for immunotherapy [20]. Sirolimus was also approved in 2015 for the treatment of lymphangioleiomyomatosis (LAM) [20].

According to the Triple Arm Study, trough levels of sirolimus should be 4-10 ng/mL [4]. Typical doses to maintain these levels are in the range of 2–4 mg per day, taken orally [29]. Encouragingly, chronic use of such doses of sirolimus for other conditions has been associated with only minimal side effects. A study reported by *Faehling *et al. illustrated the potential for long-term treatment with sirolimus [30]. A female LAM patient with a history of progressive lung failure showed an improvement of symptoms upon initiation of low therapeutic sirolimus treatment (blood levels of 5–7 ug/L). The patient remains well and shows stable lung function after 6.5 years of sirolimus initiation and having gone through two pregnancies [30]. Herein, we review the case studies reporting successful management of varying forms of sarcoidosis with sirolimus.

### Clinical Evidence of Sirolimus Use in Sarcoidosis

#### Pulmonary Sarcoidosis

*Gupta *et al*.* describe the case of a 61-year-old nonsmoking Caucasian man, diagnosed with pulmonary sarcoidosis. The patient presented with chronic cough with mediastinal and hilar lymphadenopathy associated with bilateral mid-upper lung zone-predominant peri-lymphatic nodularity showing up on chest computed tomography scanning [31]. Bronchoscopy with endobronchial ultrasound-guided transbronchial needle aspiration showed cobblestoning of the airways and non-necrotizing granulomatous inflammation [31]. He was treated over the course of 24 months with high-dose inhaled corticosteroids, bronchodilators, systemic corticosteroids, mucolytics and cough suppressants with little relief to his symptoms. Systemic corticosteroids yielded a mild reduction in cough, but symptoms recurred upon repeated attempts to taper below 15 mg/d prednisolone. An off-label trial of sirolimus began, and after 10 months on 2 mg per day, his cough largely resolved with significant improvement in peri-lymphatic nodularity [31].

#### Cardiac Sarcoidosis

*Richards *et al*.* reported the benefits of sirolimus treatment in CS patients with contraindications to corticosteroid intensification [4]. The study follows 4 patients presented with significant ongoing cardiac inflammation. Of those, two were treated with sirolimus and another two were treated with everolimus. The two patients treated with sirolimus, showed substantial improvement in cardiac inflammation on follow-up FDG-PET imaging after 6 months of treatment without corticosteroid intensification. Conversely, the two patients treated with everolimus required an augmented treatment regimen after 6 months [4]. The apparent failure of everolimus to illicit a positive response may be due to those patients having CS symptoms for much longer than the patients treated with sirolimus [4].

#### Laryngeal and Airway Involvement

Another case report describes a 15-year-old girl diagnosed with laryngeal sarcoidosis [26]. The patient presented with gradual-onset dysphonia and dysphagia, significant weight loss, fatigue, new-onset nighttime snoring, and significant supraglottic airway obstruction with swelling of both the epiglottis and arytenoids, requiring emergency tracheostomy. She was initially treated with intravenous methylprednisolone (1 g once daily) for 3 days, followed by 40 mg oral prednisolone once daily for 8 weeks. This yielded no improvement, so her regimen was switched to subcutaneous methotrexate treatment with 15 mg once weekly for 2 weeks, followed by 20 mg weekly for 12 weeks. Prednisolone was weaned to 5 mg daily. Twenty-two weeks into treatment, marginal improvement was observed, which was insufficient for decannulation. She then started an off-label trial of sirolimus at 3 mg orally once daily to maintain trough levels of 5–10 ng/mL. Within 8 weeks, a laryngoscopy demonstrated complete resolution of supraglottic swelling, allowing for decannulation. She was weaned off prednisolone completely without complication after 1 year of maintenance therapy with sirolimus [26].

#### Multi-Systemic Sarcoidosis

Sirolimus has also been shown to be effective in two related patients with aggressive, multi-systemic forms of the disease [32]. A 51-year-old male patient initially presented with diarrhoea, rectal bleeding, distended abdomen and symptoms of dizziness at 42 years of age. He was diagnosed with ‘idiopathic inflammatory bowel disease’ and prescribed antibiotics and a short course of prednisolone, which did not improve his symptoms. Five years later he developed sarcoidosis-like skin lesions. A year later he presented with HF (LVEF = 40%), increasing pulmonary distress and allergy-induced asthma with sinusitis. Lung and lymph node biopsies were positive for non-necrotizing granulomas and inflammatory infiltrates, leading to the diagnosis of sarcoidosis with cardiac involvement. Management with methylprednisolone (20 mg daily), methotrexate (15 mg/week), infliximab-abda (800 mg IV over 8 weeks) and mycophenolate mofetil (500 mg/ × 2 daily) failed to improve his symptoms and had varying adverse side effects [32].

The patient’s biological mother, when 50-years-old, presented with difficulty breathing, extreme joint pain and inflammatory skin lesions on her scalp and neck, and was diagnosed with chronic obstructive pulmonary disease (COPD) [31]. At age 62, she presented with atrial fibrillation, was prescribed dronedarone and underwent an ablation procedure. Her symptoms worsened and she presented with non-sustained ventricular tachycardia (VT), ventricular bigeminy and atrial ectopics with aberrant conduction. LV hypertrophy, mild/moderate bi-atrial enlargement as well as enlarged caliber of the main pulmonary artery were detected by MRI. Lung nodules were also seen with a reduced pulmonary diffusion capacity; thus, her diagnosis was changed to idiopathic emphysema, and she was prescribed inhaled steroids. The patient’s diagnosis was reviewed and changed to that of sarcoidosis, when her son was diagnosed with a multi-systemic form of the disease.

Both patients began sirolimus treatment, and within one month saw significant improvement in symptoms. Specifically, cardiac and pulmonary inflammation in the son resolved within 3.5 months, while his mother was able to discontinue inhaled corticosteroids within 1 month, and within 5 months, skin and joint issues had completely resolved [32].

#### Post-Transplant Sarcoidosis

Sirolimus has also been reported as an effective treatment for systemic de novo post liver-transplantation sarcoidosis in a 53-year-old man [33]. The patient was initially treated with cyclosporine monotherapy post-transplantation. However 72 months later, he presented with multi-systemic sarcoidosis, including the development of non-necrotizing granulomas in mediastinal lymph nodes, as well as in liver tissue from biopsy. Upon treatment with sirolimus with blood trough levels of 5–6 ng/mL, the patient’s condition improved dramatically [33].

#### Cutaneous Sarcoidosis

*Redl *et al*.* presented a single-centre, randomised study treating patients with persistent and glucocorticoid-refractory cutaneous sarcoidosis with sirolimus [34]. Daily topical treatment with sirolimus did not significantly improve cutaneous lesions. Systemic treatment, however, targeting trough serum concentration of 6 ng/mL resulted in clinical and histological improvement of skin lesions in 70% of the trial participants (p = 0.018). Various morphologies of cutaneous sarcoidosis, including popular, nodular, plaque, scar and tattoo-associated disease, responded to the systemic treatment with a long-lasting effect for more than 1 year after treatment had been stopped [34].

Table 1 summarizes the cases reviewed above, while Table 2 presents a qualitative summary of patient responses to sirolimus.

**Table 1:** Summary of cases reviewed

Author	Clinical Setting	Prior Therapy	mTOR inhibitor	Outcome	Ref
*Manzia* et al. (2011)	Post-transplantation de novo sarcoidosis	Cyclosporine monotherapy	Sirolimus Trough levels: 5-6 ng/mL	Significant improvement	[33]
*Faehling* et al. (2015)	LAM		Sirolimus Trough levels: 2-5ug/L	Lung function recovered and remained stable over 2 pregnancies	[30]
*Kelleher* et al. (2020)	Laryngeal Sarcoidosis	IV-methylprednisolone Oral prednisolone Subcutaneous methotrexate Methotrexate was added	Sirolimus Trough levels: 5-10 ng/mL	Significant improvement	[26]
*Gupta* et al. (2020)	Pulmonary Sarcoidosis	Inhaled corticosteroids Bronchodilators Systemic corticosteroids Mucolytics Cough suppressants	Sirolimus Dose: 2 mg/daily	Significant improvement	[31]
*Richards* et al. (2024)	CS	Corticosteroids Mycophenolate mofetil	Everolimus Dose: 0.5 mg/ × 2 daily	No improvement – everolimus discontinued, adalimumab initiated	[4]
*Richards* et al. (2024)	CS	No prior immunosuppressive treatment	Everolimus Dose: 0.5 mg/ × 2 daily	No significant improvement	[4]
*Richards* et al. (2024)	CS	Corticosteroids Azathioprine	Sirolimus Dose: 1 mg/daily	Significant improvement	[4]
*Richards* et al. (2024)	CS	No prior immunosuppressive treatment	Sirolimus Dose: 1 mg/daily	Significant improvement	[4]
*Brown* et al. (2025)	Multi-systemic sarcoidosis	Antibiotics Prednisone Albuterol Methotrexate + Rheumate Infliximab Mycophenolate mofetil	Sirolimus Dose: 4 mg/daily	Significant improvement No evidence of inflammation on PET/FDG scan	[32]
*Brown* et al. (2025)	Multi-systemic sarcoidosis	Dronedarone Inhaled steroids	Sirolimus Initial dose: 2 mg/daily Follow-up dose: 4 mg/daily	Significant improvement in inflammation, breathing, arrhythmias, joint pain and skin lesions. Further improvement upon dose increase	[32]
*Redl *et al*.* (2024)	Cutaneous sarcoidosis	Glucocorticoids	Sirolimus Trough levels: 6 ng/mL	Significant improvement in all forms of cutaneous lesios in 70% of the patients	[34]

**Table 2:** Qualitative summary table of patient response to sirolimus treatment

Clinical setting	Number of patients improved	Treatment duration	Adverse effects	Follow-up period	Ref
Post-transplantation	1/1	2 years	N/A	2 years	[33]
LAM	1/1	6.5 years	N/A	6.5 years	[30]
Laryngeal sarcoidosis	1/1	8 weeks	N/A	1 year	[26]
Pulmonary sarcoidosis	1/1	10 months	N/A	10 months	[31]
CS	2/2	6 months	N/A	6 months	[4]
Multisystemic sarcoidosis	2/2	5 months	N/A	5 months	[32]
Cutaneous sarcoidosis	7/10	4 months	N/A	> 1 year	[34]
Total	17/20	8 weeks – 6.5 years	N/A	5 months – 6.5 years	

## Discussion

Although largely effective, the drugs currently used for the management of sarcoidosis are associated with significant long-term side effects [35]. In the studies herein reviewed, sirolimus demonstrated consistent benefit across multiple sarcoidosis phenotypes, including pulmonary [31], cardiac [4], laryngeal/airway [26], multisystemic [32], post-transplant [33], and cutaneous disease [34]. Systemic dosing of 2–3 mg/day (trough 5–10 ng/mL) was typically used, with responses ranging from rapid resolution of laryngeal obstruction within 8 weeks [26], to pulmonary and cardiac improvement over 6–10 months [4, 31], and multisystemic remission as early as 1–3 months [32]. Importantly, improvements appeared to be durable, with effects in cases lasting over 1 year after discontinuation [34]. Across these cases, sirolimus was well tolerated, with no serious adverse effects reported. In contrast, within the same literature, methotrexate provided only marginal benefit in refractory laryngeal disease [26] and failed in multisystemic sarcoidosis [32]; infliximab likewise failed to control aggressive multisystemic disease, both agents being associated with variable adverse effects [32]. Azathioprine and adalimumab were not represented in these case studies, preventing direct comparison. Taken together, these accounts suggest that sirolimus may in some cases offer superior efficacy and tolerability compared with current management options.

Based on the current literature, sirolimus is best considered a second- to third-line option: it is primarily reserved for steroid-refractory or multi-systemic disease that has failed conventional immunosuppression and can also function as maintenance therapy in certain contexts [26].

We need to highlight that the evidence presented is primarily derived from individual case reports, small case series and a single-centre trial in cutaneous disease. There are no large randomized controlled trials comparing sirolimus directly with standard therapies. As a result, the relative efficacy, safety and tolerability of sirolimus currently remain uncertain. Furthermore, most reports involve heterogenous patient populations, varying dosing regimens and mostly short follow-up periods, limiting generalizability and the ability to establish long-term outcomes or optimal treatment protocols. We also acknowledge a potential publication bias, since only cases with a positive response to sirolimus are reported in the literature.

In addition to these evidence gaps, several practical challenges may affect the clinical use of sirolimus. As an off-label therapy, sirolimus requires careful documentation, patient counselling, and may face insurance coverage barriers. Cost and accessibility can also be limiting, particularly in regions without generics or where off-label prescriptions are not reimbursed. Finally, effective and safe use necessitates regular monitoring of trough serum concentrations (typically 5–10 ng/mL), which adds complexity, potential burden for patients, and risk for adverse effects if levels are not optimal [36, 37].

Although no adverse effects were reported in the studies included in this review, high doses of sirolimus (15 mg loading dose followed by 5 mg daily or higher) have been associated with increased risk of side effects. Documented toxicities at these levels include severe hyperlipidemia, thrombocytopenia, leukopenia, anemia, edema, hypertension, impaired wound healing, interstitial pneumonitis, proteinuria, and oral ulcers. This further adds to the importance of dose adjustment and plasma monitoring to balance efficacy and safety [38].

To better define the role of sirolimus in sarcoidosis, multi-centre registries and prospective phase II studies should be prioritized. Registries should systematically capture real-world data on patient demographics, organ involvement, dosing regimens, treatment duration, clinical outcomes, and adverse events, providing valuable insights into efficacy and safety across diverse populations. Phase II trials could evaluate sirolimus in corticosteroid-refractory sarcoidosis, ideally with standardized outcome measures. Biomarkers such as mTOR pathway activity in affected tissues and quantitative FDG-PET imaging should be incorporated to objectively monitor treatment response and guide dose adjustments.

## Conclusion

To conclude, herein we review case reports where sirolimus has been effective in treating multiple phenotypes of sarcoidosis. Although all patients showed remarkable improvement in response to sirolimus, no randomized controlled trials have taken place. Head-to-head comparisons between sirolimus and standard second- and third-line immunosuppressants would be instrumental in establishing its relative efficacy, safety, and optimal positioning in the treatment algorithm for corticosteroid-refractory sarcoidosis.

## Data Availability

No new data were created or analyzed in this study.

## Abbreviations

BMD: Bone mineral density
CS: Cardiac sarcoidosis
Cx43: Connexin43
EF: Ejection fraction
FKBP12: FK506-binding protein 12
FTG-PET: Fludeoxyglucose Positron Emission Tomography
HF: Heart failure
HPA: Hypothalamic-pituitary-adrenal
IV: Intravenous
LAM: Lymphangioleiomyomatosis
LVEF: Left ventricular ejection fraction
MRI: Magnetic resonance imaging
mTOR: Mammalian target of rapamycin
NFκB: Nuclear factor kappa beta
SCD: Sudden cardiac death
TNFα: Tumour necrosis factor alpha
TSC1/2: Tuberous sclerosis complex ½
VT: Ventricular tachycardia
WES: Whole-exome sequencing
